# High cell density growth of High Five suspension cells in DO-controlled wave-mixed bioreactors

**DOI:** 10.1186/1753-6561-5-S8-P123

**Published:** 2011-11-22

**Authors:** Teddy Beltrametti, Nicole C Bögli, Gerhard Greller, Regine Eibl, Dieter Eibl

**Affiliations:** 1 Institute of Biotechnology, Zurich University of Applied Sciences and Facility Management (ZHAW), Wädenswil CH-8820, Switzerland; 2 Sartorius Stedim Biotech, Göttingen, D-37075, Germany

## 

Insect cells such as High Five cells used in the manufacture of biopharmaceuticals are best grown in wave-mixed bioreactors (1). This is due to the continual blending of foam with the culture medium which results from the wave-induced mixing and permanent renewal of the medium surface. Even the addition of an antifoam agent is not required.

Process conditions which ensure maximum High Five cell densities and which have been reported to be about 8 x 10^6^ cells x mL^-1^ (2) were determined in Biostat CultiBag RM50 optical experiments for batch mode and 1 L culture volume. Seed inoculum for these experiments was generated in single-use shake flasks (Corning) incubated in an Infors`Multitron shaker (27°C, 100 rpm, 25 mm shaking diameter). Biostat CultiBag RM50 optical was controlled in four different modes: non-pH- and non-DO-controlled, DO- controlled, pH-controlled, DO- as well as pH-controlled. The DO level was guaranteed by increasing the rocking rate up to 28 rpm and, if required by addition of pure oxygen. In process control was supplemented with off-line analyses of cell density, viability, metabolites (glucose, glutamine, glutamate, lactate, ammonium) and pH.

While the influence of the type of bioreactor`s control on the maximum growth rate (0.041-0.044 h^-1^) and doubling time (15.6-17.7 h) was negligible, maximum cell densities were achieved with DO regulation (set point 50%). Maximum cell densities ranged between 8.2 and 9.4 x 10^6^ cells x mL^-1^ and represent the highest values described for High Five cells so far in the literature. They are 35% higher compared to those seen in pH-controlled and non-controlled experiments. Controlling both DO and pH level did not lead to any further improvement of cell growth i.e. the range of growth parameter values was the same as that observed in the previous experiments. For High Five cell-based biopharmaceuticals this knowledge enables optimized seed inoculum/seed train production in wave-mixed bag bioreactors.
